# Using Botulinum Toxin A for Treatment of Interstitial Cystitis/Bladder Pain Syndrome—Possible Pathomechanisms and Practical Issues

**DOI:** 10.3390/toxins11110641

**Published:** 2019-11-04

**Authors:** Jia-Fong Jhang

**Affiliations:** Department of Urology, Hualien Tzu Chi Hospital, Buddhist Tzu Chi Medical Foundation, Tzu Chi University, Hualien 97047, Taiwan; alur1984@hotmail.com; Tel.: +886-3865-1825

**Keywords:** interstitial cystitis, inflammation, treatment, Botulinum toxin

## Abstract

Treatment for patients with interstitial cystitis/bladder pain syndrome (IC/BPS) is always challenging for urologists. The main mechanism of the botulinum toxin A (BoNT-A) is inhibition of muscle contraction, but the indirect sensory modulation and anti-inflammatory effect in the bladder also play important roles in treating patients with IC/BPS. Although current guidelines consider BoNT-A injection to be a standard treatment, some practical issues remain debatable. Most clinical evidence of this treatment comes from retrospective uncontrolled studies, and only two randomized placebo-control studies with limited patient numbers have been published. Although 100 U BoNT-A is effective for most patients with IC/BPS, the potential efficacy of 200 U BoNT-A has not been evaluated. Both trigone and diffuse body BoNT-A injections are effective and safe for IC/BPS, although comparison studies are lacking. For IC/BPS patients with Hunner’s lesion, the efficacy of BoNT-A injection remains controversial. Most patients with IC/BPS experience symptomatic relapse at six to nine months after a BoNT-A injection, although repeated injections exhibit a persistent therapeutic effect in long-term follow-up. Further randomized placebo-controlled studies with a larger number of patients are needed to support BoNT-A as standard treatment for patients with IC/BPS.

## 1. Introduction

Since the early 19th century, patients who exhibited chronic bladder pain, urinary frequency, and urgency without evidence of urinary tract infection or bladder stones have been diagnosed with interstitial cystitis (IC) [1]. As our understanding of IC has progressed over the past 200 years, its name and definition have changed many times.[1] Because patients with IC may not experience bladder inflammation, the term “interstitial cystitis/bladder pain syndrome” (IC/BPS) is considered to be more suitable and is widely used in current guidelines [2,3]. IC/BPS has attracted considerable attention among urologists. A recent epidemiology study revealed that the prevalence of IC/BPS is 4.2% for the high-sensitivity definition, whereas it is 1.9% for the high-specificity definition [4]. Although the prevalence has shown an increasing trend in recent years [4], the treatment of patients with IC/BPS remains challenging for urologists. Current guidelines recommend the use of oral medications, such as amitriptyline and cimetidine, as well as intravesical instillation therapy (i.e., heparin and hyaluronic acid) to treat patients with IC/BPS [2]. However, no single treatment is effective for all patients with IC/BPS, and symptomatic relapse after the abovementioned treatments is common among patients with IC/BPS. Thus, patients with IC/BPS need a more effective treatment with greater durability.

For complicated lower urinary-tract diseases (LUTDs), treatment options are sometimes limited. Over the past two decades, biotoxins (e.g., resiniferatoxin and capsaicin) have gradually been included as possible treatment modalities for complicated LUTDs [5,6]. Botulinum toxin (BoNT), which is produced by the bacterium *Clostridium botulinum* and related species, is regarded as the most potent poisonous neurotoxin worldwide [7]. In 1988, Dykstra et al. first used a BoNT type A (BoNT-A) injection to treat patients with spinal-cord injury and detrusor–sphincter dyssynergia [8]. Today, BoNT-A is widely used to treat various types of complicated LUTDs, and many clinical trials have proven its efficacy [9]. For patients with IC/BPS, the efficacy of traditional conservative treatments is typically insufficient [2]. In recent years, the treatment of IC/BPS has shown great progress with the use of the intravesical BoNT-A injection, and laboratory studies have demonstrated bladder improvement after BoNT-A injection [10]. This review summarizes the possible pathomechanisms of using BoNT-A for treatment of IC/BPS in the bladder and examines the practical issues and long-term efficacy of such treatment.

## 2. Methods

This is a non-systemic review. The PubMed search terms including “botulinum toxin” and “interstitial cystitis” were used to find published studies. The search results were used to summarize possible mechanisms and current evidence to analyze several practical issues with the use of BoNT-A in treating IC/BPS. The results were generated based on the author’s knowledge and clinical experience in this field; hence, they were highly impacted by the author’s bias in selecting and interpreting studies.

## 3. Results

### 3.1. Possible Pathomechanisms for BoNT-A in Treating IC/BPS

BoNT is well-known for its ability to inhibit acetylcholine release, which results in muscle paralysis [11]. However, current evidence shows that the possible pathomechanisms of using BoNT-A for treatment of IC/BPS involve additional pathways, including anti-inflammatory effects and sensory modulation in the urothelium [12].

#### 3.1.1. Inhibition of Detrusor Muscle Activity

BoNT-A consists of a 50 kDa light chain and a 100 kDa heavy chain connected by a weak disulfide bond [13]. In presynaptic nerve endings, the C-terminal of the heavy chain binds to the synaptic vesicle protein 2 on the neuronal cell membrane, enabling BoNT-A to be internalized within the nerve terminal by endocytosis [14]. Although the heavy chain facilitates the entrance of BoNT-A into neurons, the light chain is the biologically active moiety of BoNT-A. Synaptosome-associated protein 25 facilitates intracellular vesicle docking and membrane fusion, and is involved in the exocytotic release of neurotransmitters during synaptic transmission [15]. The light chain of BoNT-A cleaves apart synaptosome-associated protein 25 in presynaptic neurons, thereby inhibiting the release of the acetylcholine neurotransmitter by disrupting vesicular fusion with the neuronal cell membrane, resulting in flaccid muscle paralysis [11]. A previous study showed that BoNT-A could attenuate bladder contractility. In a clinical study of intravesical injection of BoNT-A 200 U in patients with neurogenic detrusor overactivity, the maximum detrusor pressures during filling cystometry were significantly reduced at the four-week follow-up [16]. Recently, abnormal high tension or spasm in smooth muscle has been suggested to be a potential mechanism for the development of chronic visceral organ pain [17]. Tension-sensitive nerve endings in the smooth muscle of visceral organs may respond to luminal distension or stretching. Mechanotransduction of low-threshold afferent nerves is associated with transient receptor potential vanilloid receptor 1 (TRPV1) activation and resulting visceral pain [17]. Although electrophysiological evidence of this phenomenon in the human bladder is lacking, patients with IC/BPS commonly exhibit a tender bladder with “spasm-like” sensations [18]. Relaxation of bladder-muscle tension might be the mechanism by which BoNT-A treats bladder pain in patients with IC/BPS. Furthermore, although the core symptom of IC/BPS is bladder pain, most patients with IC/BPS also exhibit urinary frequency, urgency, and incontinence. Our previous study showed that detrusor overactivity could be detected in approximately 10% of patients with IC/BPS [19]. BoNT-A injection-induced inhibition of detrusor muscle overactivity could relieve frequency and urgency symptoms, thus improving the quality of life for patients with IC/BPS.

#### 3.1.2. Sensory Modulation in the Urothelium

Generally, patients with botulism do not lose sensory nerve function. However, intravesical BoNT-A injection results in sensory modulation, both in pain reduction and urgency reduction in patients with IC/BPS or an overactive bladder [2,9]. Recent laboratory evidence revealed that BoNT-A injection indeed could alter the expression levels of sensory neurotransmitters and receptors in the bladder. Substance P and calcitonin gene-related peptide are small peptides that act as neurotransmitters in nociception [20,21]. In rats with cyclophosphamide-induced cystitis, the intravesical BoNT-A injection has been shown to significantly reduce the bladder expression levels of substance P and calcitonin gene-related peptide [22]. Glutamate is an excitatory neurotransmitter that plays a crucial role in central and peripheral pain pathways, along with its receptor [23]. BoNT-A injection induced significant downregulation of glutamate expression levels in human and rat skin [24,25]. In the bladder, adenosine 5’-triphosphate acts on the purinergic receptor, serving as the signal for the central nervous system to perceive bladder fullness [26]. In animal studies, BoNT-A intravesical injection was found to inhibit adenosine 5’-triphosphate release from rat bladder urothelium [27,28]. TRPV1 is a vanilloid receptor that is expressed in nociceptive afferent neurons, which can be activated by heat, protons, or vanilloid chemicals (e.g., capsaicin and resiniferatoxin) [29,30]. TRPV1 plays an important role in neural transmission in the pain pathway, and the upregulation of TRPV1 in certain diseases is accompanied by elevated pain [29,30]. In a clinical study of the use of intravesical BoNT-A injection in patients with an overactive bladder, a significant reduction of TRPV1-positive nerves was detected in bladder biopsy specimens [31]. Detrusor BoNT-A injections also led to significant reductions in the expression levels of the bladder muscarinic receptors M2 and M3, as well as the purinergic receptors P2X2 and P2X3, in patients with neurogenic detrusor overactivity [31,32]. Modulation of sensory neurotransmitters and their receptors in the bladder therefore constitutes an important mechanism by which BoNT-A can treat IC/BPS. In addition, the modulation of central nervous system sensory receptors in peripheral organs after BoNT-A injection has been observed in animal studies. The reduction of c-fos and calcitonin gene-related peptide expression levels in the dorsal horn of the spinal cord after pawl BoNT-A injection has been reported in association with the reduction of mechanical allodynia [33,34]. In a recent study, the injection of radiolabeled BoNT-A into the bladder caused retrograde transport to lumbosacral dorsal root ganglia [35]. Theoretically, intravesical injection of BoNT-A might change sensory receptor expression levels in the spinal cord, but further studies are needed to confirm this hypothesis. Although the Botulinum toxin does not directly block neurotransmitters released in the afferent nerve system, current clinical and laboratory evidence suggests that it indirectly modulates the sensory function in the bladder with pathological changes.

#### 3.1.3. Anti-Inflammatory Effect in the Urothelium

Although some histopathology studies have reported contrary evidence, most experts and guidelines currently consider bladder inflammation to be present in patients with IC/BPS—bladder inflammation is presumed to play a central role in the pathogenesis of IC/BPS [2,36,37,38]. The infiltration of mast cells and lympho-plasmacytic cells is increased in the lamina propria and detrusor muscle of the bladder in patients with IC/BPS [37,38,39]. Immunohistochemical studies also have shown increased mast-cell activity and upregulation of pro-inflammatory cytokines in the bladders of patients with IC/BPS, including inducible nitric oxide synthase, interleukin-6 (IL-6), and IL-17A [40,41]. In urine and serum from patients with IC/BPS, inflammatory cytokines (e.g., IL-1β, IL-6, IL-8, tumor necrosis factor-α, and C-reactive protein) are reportedly elevated [42,43]. Although BoNT-A is well-known for its neurotransmitter-blocking effect, there is increasing evidence that BoNT-A can inhibit the release of inflammatory cytokines in different organs. In an in-vitro study, bladders were harvested from rats with acute or chronic cystitis, then incubated in organ baths containing BoNT-A or a vehicle [22]. Reduced substance-P release in the bladder was detected in models of both acute and chronic cystitis. In another in-vivo study, BoNT-A intravesical injection was shown to reduce histological inflammation in the bladders of rats with cyclophosphamide-induced cystitis [44]. Cyclooxygenase 2 and prostaglandin E2 receptor expression levels in the bladder also were reduced in the BoNT-A injection group [44]. In the human bladder, a single BoNT-A injection does not seem to reduce bladder inflammation in patients with neurogenic detrusor overactivity, but may improve bladder fibrosis [45]. In patients with IC/BPS, our previous study showed that repeated BoNT-A injections could significantly reduce the numbers of activated mast cells in the bladder, whereas a single injection could not [46]. The vascular endothelial growth-factor expression level and apoptotic cell count in the bladder were also reduced in patients with IC/BPS after repeated BoNT-A injections; however, these levels remained significantly higher than those of the controls [41]. Additionally, urothelial barrier-function impairment is a possible pathogenic mechanism in IC/BPS [47]. Improvement in urothelial barrier function might also be a possible mechanism by which BoNT-A is used for treatment of IC/BPS. Urothelial barrier-function recovery might contribute to improvement in suburothelial inflammation. Our previous study revealed that repeated BoNT-A injections could improve the expression levels of the urothelial barrier function protein, E-cadherin, and the tight junction protein, zonula occludens-1, in patients with neurogenic detrusor overactivity [48]. However, to the best of our knowledge, the effect of BoNT-A injection on the expression levels of urothelial barrier-function proteins in patients with IC/BPS has not yet been reported.

### 3.2. The Practical Issues with the Use of BoNT-A in Treating IC/BPS

Although BoNT-A is now widely used to treat IC/BPS, it still is a novel treatment that has only been included in guidelines since 2015 [2]. Clinical evidence is still relatively limited, and several technique details of BoNT-A injection to IC/BPS patients remain unclear. The following sections list the practical issues that should be noticed by clinicians, and summarize current evidence of the debatable issues.

#### 3.2.1. Clinical Efficacy of BoNT-A in Treating IC/BPS

As noted previously, the treatment of patients with IC/BPS remains challenging for urologists. Because the pathogenesis of IC/BPS is not well-understood, it is rare for complete resolution of IC/BPS to occur after a single treatment [1]. Currently, most treatments for IC/BPS mainly target symptomatic relief, rather than resolution of disease; however, even satisfactory improvement of symptoms may not be achieved in all patients [49]. Researchers have used BoNT-A for treatment of patients with IC/BPS in clinical trials since 2004, and the initial results of various studies showed promising outcomes [50,51]. In 2004, Smith et al. first used submucosal injection of BoNT-A in the bladder trigone and floor, combined with cystoscopic hydrodistention. Among the 13 female patients in that study, the Interstitial Cystitis Problem and Symptom Index scores improved by 69% and 71% respectively; the pain, assessed by a visual analog scale, also decreased by 79%. In our pilot study, we used BoNT-A suburothelial injection into 20 sites alone to treat patients with IC/BPS for whom conventional treatments had failed [51]. The bladder visual analog scale pain score, functional bladder capacity, and daytime urinary frequency all showed mild but significant improvement at three months after treatment. More recently, many studies have shown the clinical efficacy of BoNT-A injection in treatment of IC/BPS. New double-blind, randomized controlled studies revealed that BoNT-A intravesical injection could relieve symptoms in patients with IC/BPS; these included reduced bladder pain and increased capacity [52,53,54]. BoNT-A injection is considered to be standard therapy in the American Urology Association (AUA) guidelines for IC/BPS [2], and is widely used among urologists. There are three types of BoNT-A available for treatment of patients with IC/BPS, including Botox® (onabotulinum toxin A, Allergan, Inc., CA), Dysport® (abobotulinum toxin A, Ipsen, Inc., UK), and LANTOX® (CBTX-A, Lanzhou Institute of Biological Products, China) [50,51,55]. However, symptom relapse is common after BoNT-A injection in patients with IC/BPS, and most patients require repeated BoNT-A injections [56]. The use of BoNT-A for treatment of IC/BPS has several remaining practical issues that should be addressed.

#### 3.2.2. Two Randomized, Double-Blinded, Placebo-Controlled Studies of the Use of BoNT-A Injection for Treatment of IC/BPS

Although there have been more than 50 clinical studies of the use of intravesical BoNT-A injection for treatment of patients with IC/BPS, clinicians should note that only two of those studies were randomized, double-blinded, and placebo-controlled [53,54,57]. In our first double-blinded study, we enrolled 60 patients with IC/BPS who were refractory to conventional treatments, and used normal saline intravesical injection as the placebo control [53]. At week 8, patients who received BoNT-A 100 U had significantly greater pain reduction than patients who received normal saline (−2.6 ± 2.8 vs. −0.9 ± 2.2, *P* = 0.021) [53]. The overall success rates (global response assessment ≥1) were also higher in the BoNT-A group (26/40, 63%) than in the normal saline group (3/20, 15%, *P* = 0.028). The adverse event rate was similar between the two groups. Pinto et al. published the second double-blinded study in 2018, in which 19 patients with IC/BPS were randomly assigned to intratrigonal injection of BoNT-A 100 U or normal saline [54]. At week 12, a significantly greater reduction of pain was observed in the BoNT-A group than in the normal saline group (−3.8 ± 2.5 vs. −1.6 ± 2.1, *p* < 0.05). O’Leary–Sant score reduction and quality of life improvement were also superior in the BoNT-A injection group. Notably, improvement of bladder symptoms was observed in the placebo group in both studies, which suggested that the placebo effect was strong in clinical treatment of patients with IC/BPS. The patient-inclusion criteria was also different between the two studies. Only a total of 79 patients with IC/BPS had been enrolled in randomized and placebo-controlled studies; [53,54] therefore, additional studies with high levels of evidence are necessary to confirm the therapeutic efficacy of BoNT-A injection. Recruiting more IC/BPS patients is necessary to analyze the therapeutic effect in the subgroup patients (e.g., classification with glomerulation grade).

#### 3.2.3. Dose of BoNT-A Injection in IC/BPS

The dose of BoNT-A injection for treatment of patients with LUTDs is difficult to determine. Although mortality has not been reported following medical use of BoNT-A injection, the estimated lethal dose is 2800 U in humans [58]. Most urologists and studies have established doses based on previous experience, rather than on solid supporting evidence. Previous studies demonstrated a dose-response relationship in the use of BoNT-A to treat patients with detrusor overactivity, and showed that bladder-capacity improvement was also dose-dependent [59]. Moreover, residual bladder volume increased with an increasing BoNT-A dose [59]. For patients with neurogenic detrusor overactivity, the therapeutic effects of BoNT-A 200 U and BoNT-A 300 U were similar [60]. However, dose-comparison studies for patients with IC/BPS have not been reported. Initially, several studies reported satisfactory outcomes of BoNT-A 200 U for treatment of patients with IC/BPS [50,51,61,62,63], particularly those studies by the Giannantoni group in Italy [61,62]. Our initial experience with a very limited number of patients suggested that BoNT-A 200 U may have a better therapeutic effect than 100 U injection, but this was not found to be statistically significant [64]. However, the rates of dysuria and high residual-urine volume also seemed to be higher in the 200 U group. Most studies have used 100 U BoNT-A for patients with IC/BPS, and this dose is widely used in clinical practice. The American Urology Association guidelines suggest that the use of BoNT-A 100 U is an appropriate treatment and may substantially reduce morbidity compared to BoNT-A 200 U [2]. However, for refractory patients with IC/BPS who do not have satisfactory responses to BoNT-A 100 U, a 200 U injection might be a reasonable second-choice treatment option. The potential therapeutic effect of BoNT-A 200 U injection should be investigated in further dose-comparison studies.

#### 3.2.4. Location of Bladder Injection: Trigone or Bladder Body

Most studies have used BoNT-A bladder-body injection to treat patients with bladder disease [65]. For the inhibition of bladder contractility and associated adverse effects, some researchers have used BoNT-A injection in the trigone and bladder floor, rather than the entire bladder body, for patients with IC/BPS [54,61,62,66]. The trigone has been regarded as the sensory input center in the bladder since the 1970s [67]. Neurophysiology analysis revealed that the trigone contains significantly higher levels of adrenergic and muscarinic nerve innervation relative to the bladder body [67]. A recent three-dimensional image reconstruction analysis of the nerve further demonstrated that bladder autonomic innervation is concentrated in the bladder trigone [68]. The injection of BoNT-A in the bladder trigone to block nociceptive neurotransmitters is a reasonable procedure, and has the potential to avoid inhibition of bladder contractility. However, the mechanisms of IC/BPS pathogenesis include abnormal bladder sensory input, as well as active inflammation in the bladder body. The anti-inflammatory effect of BoNT-A trigone injection may be inferior to that of diffuse bladder-body injection. Our previous study compared BoNT-A injection in the bladder body with that of injection in the trigone in patients with idiopathic detrusor overactivity [69]. There were no significant differences in treatment success rates or changes in urgency severity score, and the incidence of adverse events was similar between trigone and bladder-body injections. Another animal study used BoNT-A injection on one side of the bladder and found that synaptosome-associated protein 25 was cleaved on the opposite side of the bladder [70]. The findings of that study demonstrate that BoNT-A injection could spread across the guinea pig bladder; this phenomenon also may occur in the human bladder. Theoretically, trigone and bladder body BoNT-A injections might have similar therapeutic effects, but comparison studies are needed to confirm this hypothesis. For elderly IC/BPS patients with inadequate bladder contractility, the use of BoNT-A trigone injection appears to be reasonable.

#### 3.2.5. Long-Term Therapeutic Effects of BoNT-A in IC/BPS

BoNT turnover in neurons is primarily mediated by the lysosomal/autophagic and ubiquitin-proteasome systems [71]. Most studies have shown persistent muscle paralysis in humans for four to six months following treatment with BoNT-A [72,73]. The persistence of the therapeutic effect after a single BoN-A injection in patients with LUTDs has varied among studies. In a study comparing the different types of BoNT-A injection for patients with neurogenic detrusor overactivity, approximately half of the patients (53.4%) exhibited continence at three months postinjection [74]. The mean interval of on-demand repeated injection was eight months, and no significant differences were found among types of BoNT-A injections. When assessing the therapeutic effect of a single BoNT-A injection in patients with IC/BPS, the longest follow-up duration in published studies is currently one year [52,62]. In 2008, Giannantoni et al. reported significant symptomatic relapse at five months after 200 U BoNT-A injection in the trigone and lateral wall, in terms of bladder pain, urinary frequency, and bladder capacity [62]. All patients exhibited recurrent pain at 12 months postinjection. In 2015, Akiyama et al. used 100 U BoNT-A trigone injections and reported similar results.[52] The response rates (global response assessment ≥+1: slightly improved) were 38.2% at six months postinjection and 20.6% at 12 months postinjection [52]. Although BoNT-A injection was effective for symptomatic relief in IC/BPS, remission was common and repeated injection was necessary for most patients.

Because symptomatic relapse is common, the persistence of therapeutic effects after repeated BoNT-A injection is an important issue. Evidence from cosmetic use of BoNT-A showed that treatment failure may occur after repeated injections [52,75]. The formation of neutralizing antibodies after repeated BoNT-A injections could include antibodies that target the functionally active part of BoNT-A, thereby leading to treatment failure [76]. In a human study, the formation of neutralizing antibodies after Botox® injection was observed in an estimated 5–15% of patients [77]. In cosmetic use, potential risk factors for development of neutralizing antibodies included a single injection of >200 U and repeated injection within one month [75]. For BoNT-A injection in patients with LUTDs, current evidence suggests that the therapeutic effect could persist after repeated injections. Kennelly et al. performed a prospective study of 240 neurogenic detrusor overactivity patients who underwent up to six repeated BoNT-A injections within a four-year period [60]. Symptomatic improvement among injections did not reach statistical significance, particularly in terms of incontinence episodes and voided volume. For patients with IC/BPS, studies also showed that repeated BoNT-A injections could provide persistent symptomatic relief [78,79,80]. Notably, beginning in 2006, we performed a BoNT-A injection of 100 U every six months in patients with refractory IC/BPS until they wished to discontinue treatment [78]. Significant symptomatic improvement, including to bladder pain, O’Leary–Sant scores, and global response assessment, persisted after the fourth BoNT-A injection. Within a follow-up period of up to 79 months, patients who received repeated injections had a better success rate and longer therapeutic duration than those who received a single injection. The rate of adverse effects did not increase with the number of BoNT-A injections. Therefore, repeated BoNT-A injections may be both effective and safe for patients with IC/BPS.

However, one study detected serum BoNT-A antibodies in patients who received bladder BoNT-A injections, and the presence of such antibodies was associated with treatment failure [81]. Although repeated BoNT-A injections in the bladder are generally effective, the formation of neutralizing antibodies might cause treatment failure in some patients. Further study is necessary to investigate whether the neutralizing antibodies are present in the bladder after repeated BoNT-A injection.

#### 3.2.6. BoNT-A Injection for IC/BPS with Hunner’s Lesion

Hunner’s lesion is a specific cystoscopic finding that is characterized by a circumscript, reddened mucosa with small vessels radiating toward a central scar [82]. Patients with IC/BPS and Hunner’s lesion (HIC) typically experience significant sharp bladder pain and require cystoscopic bladder electrocauterization [1,82]. The efficacy of BoNT-A injection in HIC is controversial. Our study used diffuse bladder-body injection of BoNT-A in patients with HIC; notably, these patients showed no significant changes in any clinical or urodynamic variables [83]. Another study reported that the efficacy of BoNT-A injection in patients with HIC was not superior to treatment with hydrodistention alone [84]. In contrast, Pinto et al. compared the treatment outcomes of intratrigonal BoNT-A injection between patients with HIC and patients with IC/BPS without Hunner’s lesion [85]. Both groups had equal responses to BoNT-A, as well as significant improvement that included lessening of bladder pain, urinary frequency, and O’Leary–Sant scores. In another study, complete remission of Hunner’s lesion was observed in three of five patients with HIC after BoNT-A injection [86]. All of the abovementioned studies were limited to small numbers of patients (≤0 patients with HIC per study), and the results were conflicting among studies. In the past, the definition of Hunner’s lesion might have differed among countries [82]; hence, the characteristics of patients with HIC included in previous studies may lack consistency. In the study by Pinto, the patients with HIC and patients with IC/BPS without Hunner’s lesion had similar symptom severity at baseline; in contrast, the patients with HIC in our study had much more severe baseline symptoms. From our perspective, BoNT-A injection alone is insufficient for satisfactory symptomatic relief in patients with severe HIC. Further studies with larger numbers of patients and clear definitions of Hunner’s lesion are necessary.

## 4. Conclusions

Intravesical BoNT-A injection provides a promising option in treating IC/BPS. Randomized placebo-controlled trials have revealed that intravesical BoNT-A injection is effective for relief of pain and urinary symptoms. The mechanisms by which BoNT-A is effective for treatment of IC/BPS include inhibition of detrusor muscle activity, as well as directly sensory modulation and inflammation control in the urothelium. Although current guidelines consider BoNT-A injection to be the standard treatment, some practical issues remain. Most clinical evidence comes from retrospective uncontrolled studies, and there are only two randomized, placebo-controlled studies with limited patients numbers to support BoNT-A efficacy in IC/BPS. Most patients with IC/BPS experience symptom relapse at six to nine months after BoNT-A injection, although repeated BoNT-A injections have persistent therapeutic effects. The optimal BoNT-A injection dose and site have not been well-investigated in comparison studies. For the IC/BPS patients with Hunner’s lesion, clinical efficacy is still unclear. Further high-level evidence studies with greater numbers of patients are necessary to support using BoNT-A injection as a standard treatment for patients with IC/BPS.
